# An invasive mole with pulmonary metastases in a 55-year-old postmenopausal Syrian woman: a case report and review of the literature

**DOI:** 10.1186/s13256-020-02630-3

**Published:** 2021-01-18

**Authors:** Sawsan Ismail, Karen Mikhael, Nehad Salloum, Zuheir Alshehabi

**Affiliations:** 1 Department of Pathology, Cancer Research Center, Faculty of Medicine, Tishreen University, Lattakia, Syria; 2 Faculty of Medicine, Tishreen University, Lattakia, Syria; 3 Department of Obstetrics and Gynecology, Tishreen University Hospital, Lattakia, Syria; 4 Department of Pathology, Cancer Research Centre, Faculty of Medicine, Tishreen University, Lattakia, Syria

**Keywords:** Invasive mole, Gestational trophoblastic neoplasms, Postmenopausal woman, Pulmonary metastases

## Abstract

**Background:**

Invasive mole is a subtype of gestational trophoblastic neoplasms (GTNs) that usually develops from the malignant transformation of trophoblastic tissue after molar evacuation. Invasive moles mostly occur in women of reproductive age, while they are extremely rare in postmenopausal women.

**Case presentation:**

We present the case of a 55-year-old postmenopausal Syrian woman who was admitted to the emergency department at our hospital due to massive vaginal bleeding for 10 days accompanied by constant abdominal pain with diarrhea and vomiting. Following clinical, laboratory and radiological examination, total hysterectomy with bilateral salpingo-oophorectomy was performed. Histologic examination of the resected specimens revealed the diagnosis of an invasive mole with pulmonary metastases that were diagnosed by chest computed tomography (CT). Following surgical resection, the patient was scheduled for combination chemotherapy. However, 2 weeks later the patient was readmitted to the emergency department due to severe hemoptysis and dyspnea, and later that day the patient died in spite of resuscitation efforts.

**Conclusion:**

Although invasive moles in postmenopausal women have been reported previously, we believe our case is the first reported from Syria. Our case highlights the difficulties in diagnosing invasive moles in the absence of significant history of gestational trophoblastic diseases. The present study further reviews the diagnostic methods, histological characteristics and treatment recommendations.

## Introduction

Invasive mole is defined as a subtype of gestational trophoblastic neoplasms (GTNs) characterized by the presence of edematous trophoblastic tissue and hydropic chorionic villi invading the myometrium with or without vascular and extrauterine invasion. GTNs represent a heterogeneous group of pregnancy-related tumors including choriocarcinoma, placental cell tumor, and invasive moles [1]. Invasive moles mostly occur in women of reproductive age, while they are extremely rare in postmenopausal women, with only a few cases reported in the literature [2]. Furthermore, the incidence of invasive moles varies geographically, with the highest rate in the South-East Asian Region (SEAR) and the Middle East countries, while the lowest rate is reported from Europe and North America [2]. Herein, we report a unique and challenging case of an invasive mole with pulmonary metastases in a postmenopausal Syrian woman.

## Case presentation

We report the case of a 55-year-old postmenopausal Syrian woman who presented to the emergency department at our hospital with a history of massive vaginal bleeding for 10 days accompanied by constant abdominal pain with diarrhea and vomiting. Her obstetrical history reported natural menopause 15 months earlier and a history of 14 normal vaginal deliveries (G14P14A0), with the last delivery being 13 years before the current presentation. She had no history of spontaneous abortions, contraceptive drug use or molar pregnancies. Medical and family history were unremarkable.

**Fig. 1:**
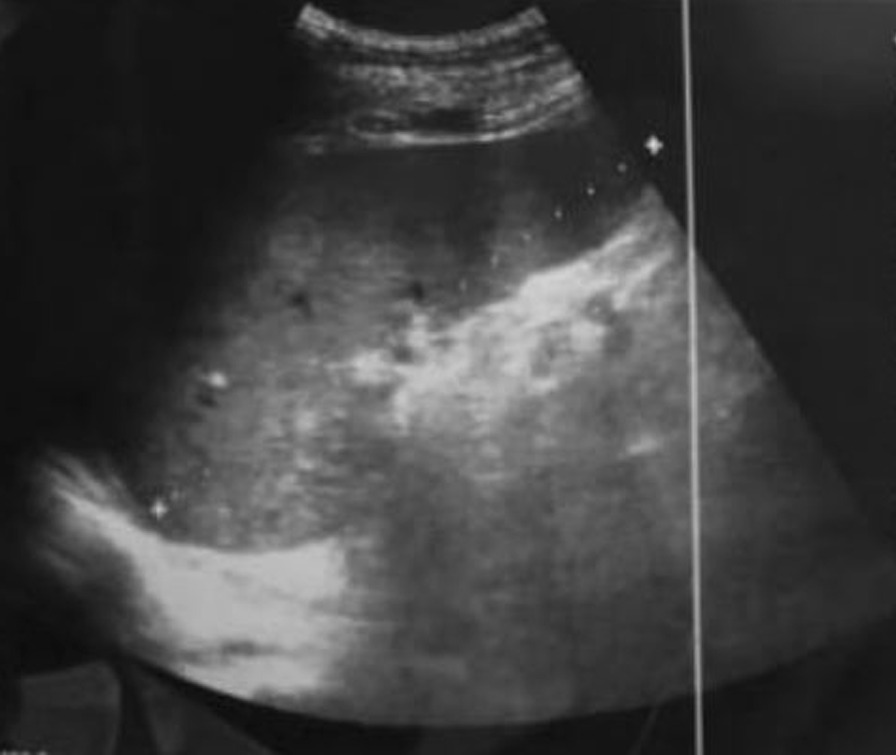
Pelvic ultrasound demonstrating an enlarged uterus with a heterogeneous mass obliterating the endometrial cavity, with a vesicular appearance

Physical examination revealed a palpable pelvic mass extending up to approximately 3 cm above the umbilicus. The serum beta-human chorionic gonadotropin (b-HCG) level was determined to be 542.250 mU/mL. Pelvic ultrasonography demonstrated an enlarged uterus the size of 24-week gestation, with a heterogeneous mass obliterating the endometrial cavity, with a vesicular appearance (Fig. 1) and normal ovaries. Computed tomography (CT) scan of the abdominopelvic region confirmed the presence of a well-demarcated mass measuring 25 × 20 × 13 cm with very high-density central cystic content. Thus the primary differential diagnosis included a metastatic endometrial leiomyosarcoma, a choriocarcinoma and an invasive mole. Chest and cranial CT scans were also performed to detect possible metastases, demonstrating mild bilateral pleural effusion with multiple nodular lesions in the basal lung segments, whereas no lesions were detected elsewhere. As the patient was a postmenopausal woman with massive vaginal bleeding, the surgical decision was to perform total hysterectomy with bilateral salpingo-oophorectomy.

**Fig. 2:**
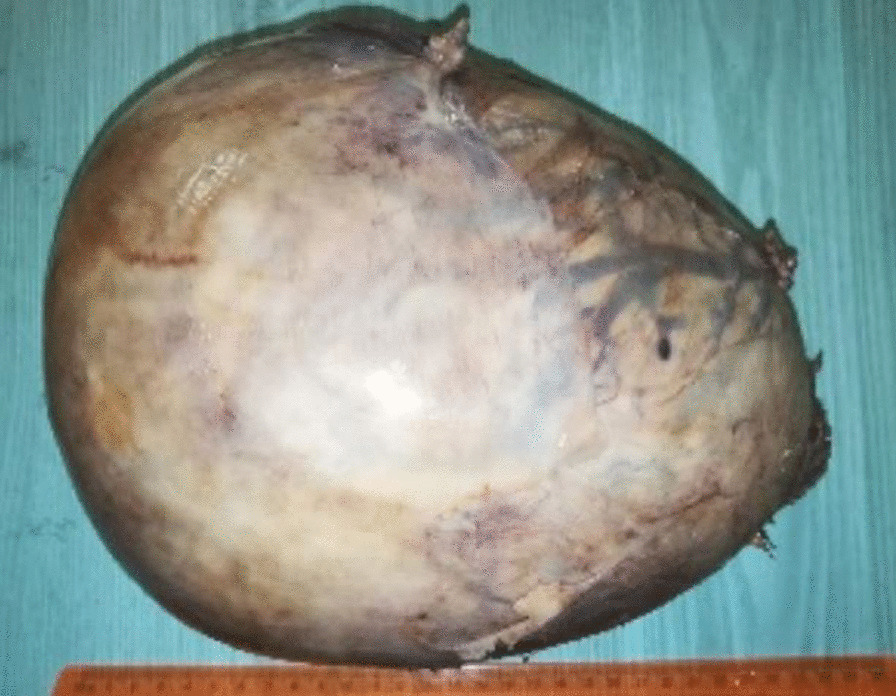
A macroscopic image of the resected mass measuring 25 × 20 × 13 cm

**Fig. 3:**
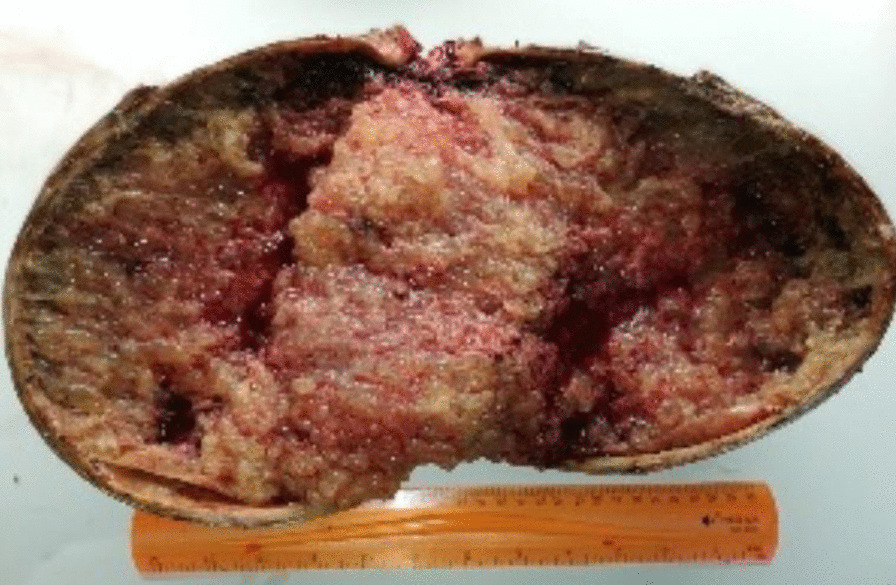
A macroscopic image of the endometrial cavity which was filled with hemorrhagic villi and edematous grape-like vesicles

**Fig. 4:**
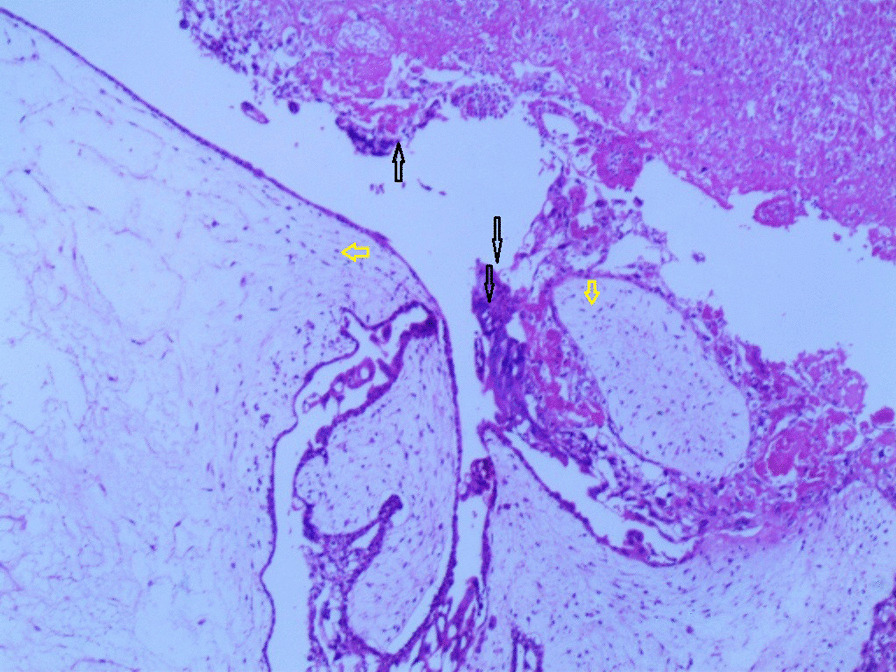
A microscopic image demonstrating a circumferential proliferation of abnormal hyperchromatic trophoblastic cells (black arrows) surrounding edematous hydropic villi (yellow arrows) (hematoxylin and eosin [H&E] stain, original magnification ×40)

**Fig. 5:**
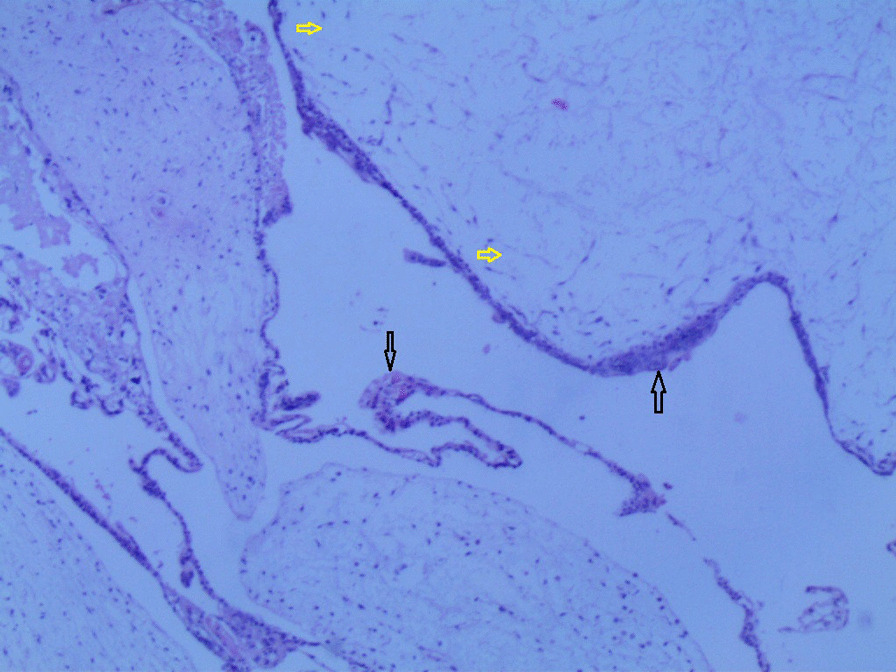
A microscopic image demonstrating edematous hydropic villi (yellow arrows) surrounded by abnormal trophoblastic cells (black arrows) (hematoxylin and eosin [H&E] stain, original magnification ×40)

**Fig. 6:**
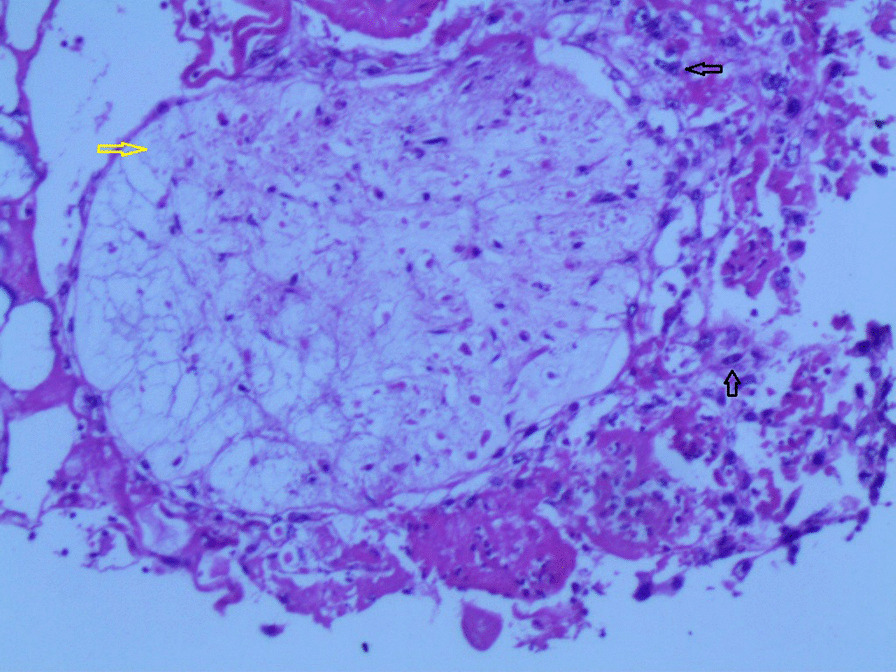
A microscopic image with higher magnification demonstrating an edematous hydropic villus (a yellow arrow) surrounded by abnormal hyperchromatic trophoblastic cells (black arrows) (hematoxylin and eosin [H&E] stain, original magnification ×100)

**Fig. 7:**
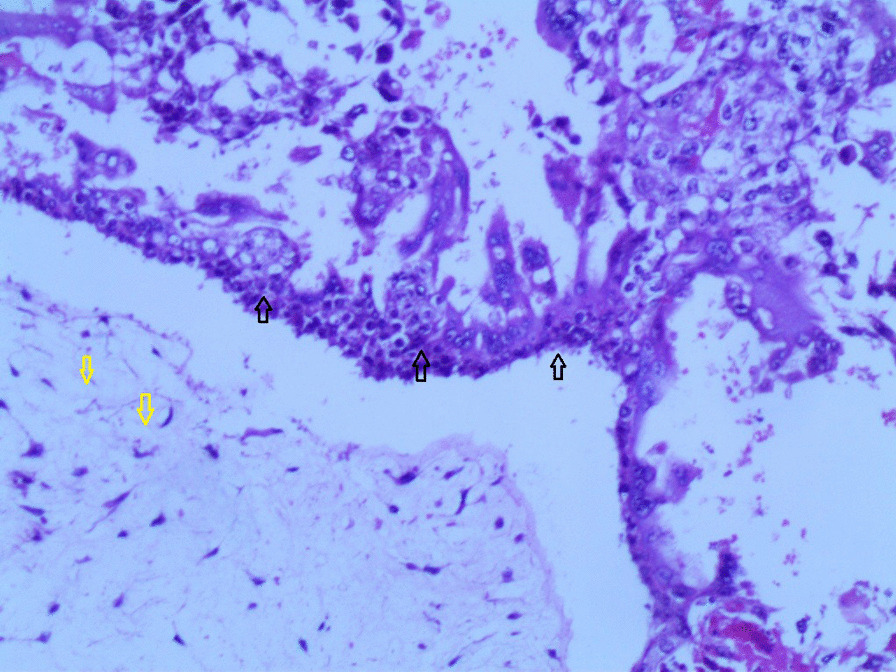
A microscopic image with higher magnification demonstrating a circumferential proliferation of abnormal hyperchromatic trophoblastic cells (up to the right: black arrows) surrounding edematous hydropic villi (down to the left: yellow arrows) (hematoxylin and Eosin [H&E] stain, original magnification ×100)

**Fig. 8:**
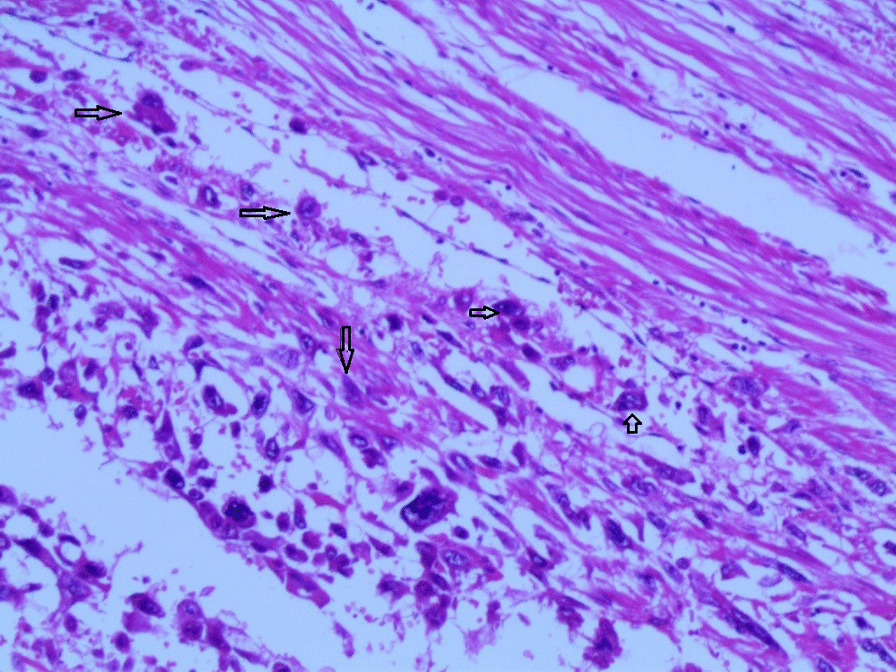
A microscopic image demonstrating the abnormal trophoblastic cells (black arrows) invading the myometrium (hematoxylin and eosin [H&E] stain, original magnification ×100)

**Fig 9:**
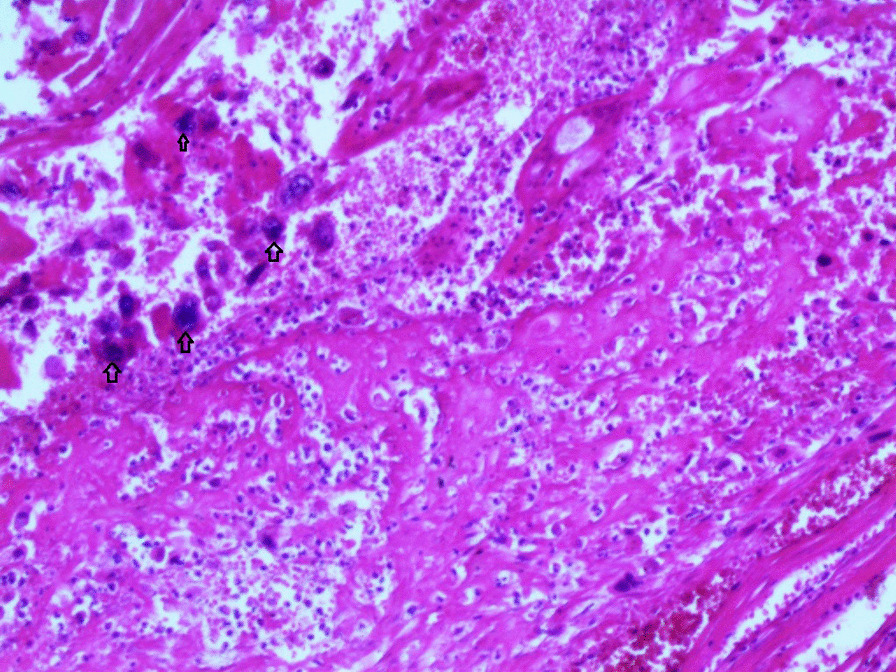
A microscopic image demonstrating the abnormal trophoblastic cells (black arrows) invading the myometrium with areas of necrosis and hemorrhage (hematoxylin and eosin [H&E] stain, original magnification ×100)

**Fig 10:**
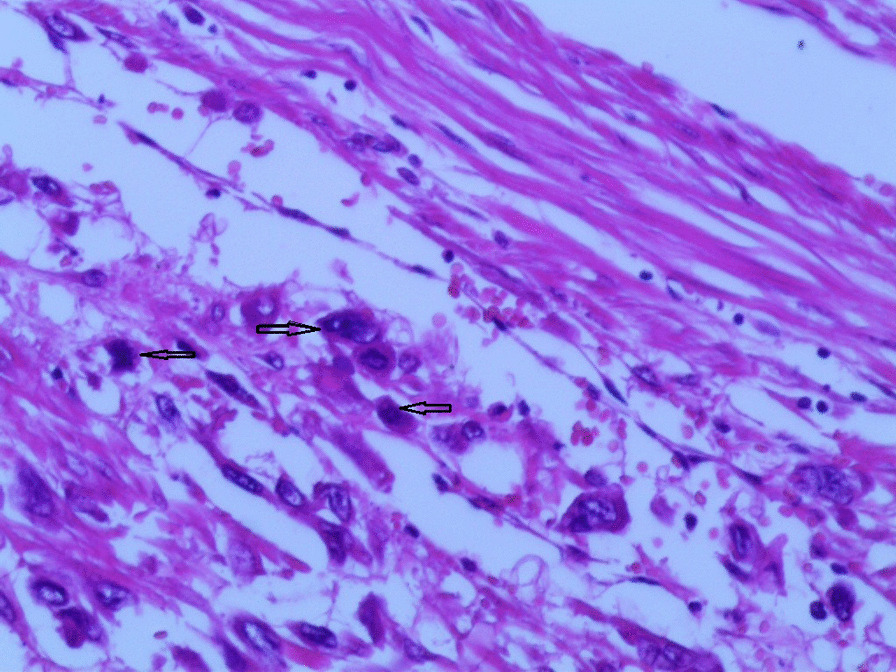
Microscopic images with higher magnification demonstrating the abnormal trophoblastic cells (black arrows) invading the myometrium (hematoxylin and eosin [H&E] stain, original magnification ×200)

**Fig 11:**
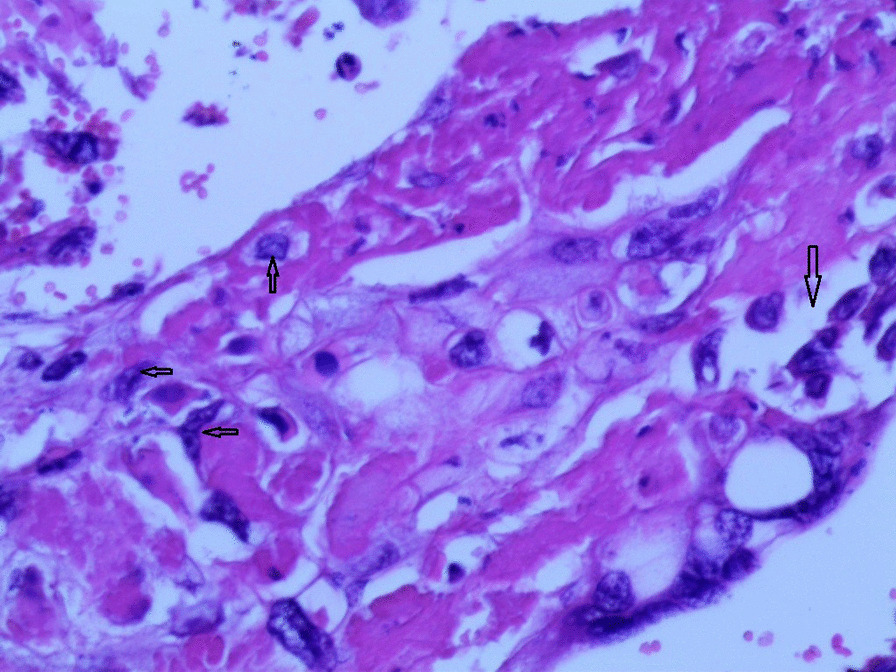
Microscopic images with higher magnification demonstrating the abnormal trophoblastic cells (black arrows) invading the myometrium (hematoxylin and eosin [H&E] stain, original magnification ×200)

**Fig. 12:**
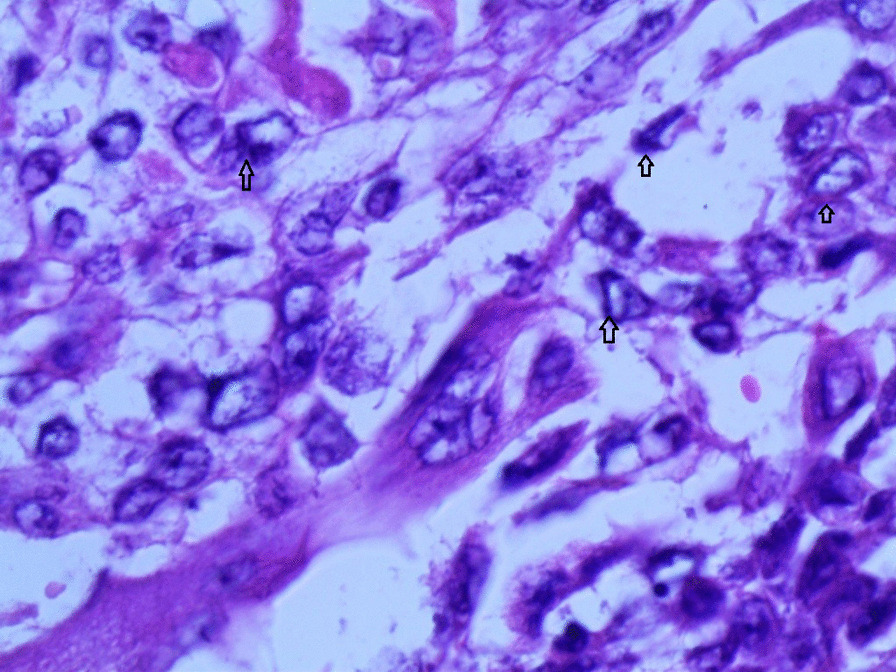
A microscopic image with high magnification demonstrating the atypical trophoblastic cells (black arrows) (hematoxylin and eosin [H&E] stain, original magnification ×400)

**Fig. 13:**
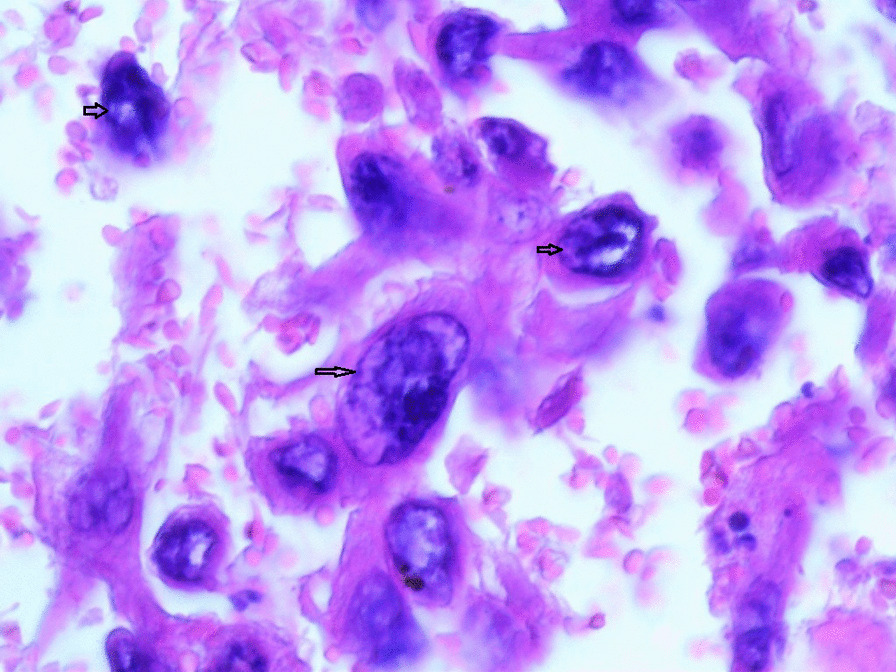
A microscopic image with high magnification demonstrating the atypical trophoblastic cells (black arrows) (hematoxylin and eosin [H&E] stain, original magnification ×600)

Gross examination revealed an enlarged uterus measuring 25 × 20 × 13 cm and weighing 3350 g, (Fig. 2), with normal bilateral fallopian tubes and ovaries. The endometrial cavity was highly enlarged, and filled with hemorrhagic villi and edematous grape-like vesicles measuring up to 1.5 cm in diameter (Fig. 3). Microscopic examination demonstrated a circumferential proliferation of abnormal hyperchromatic trophoblastic cells surrounding edematous hydropic villi invading the myometrium, with a few scattered trophoblastic cells within blood vessels (Figs. 4, 5, 6, 7, 8, 9, 10, 11, 12, 13).

Based on detailed observation of morphological features accompanied by clinical and radiological correlation, the diagnosis was confirmed as an invasive mole with pulmonary metastases and classified as high risk (stage III: score 14) according to the International Federation of Gynecology and Obstetrics (FIGO) staging system and World Health Organization (WHO) prognostic scoring system: (FIGO III: disease in the lungs, score 14: age > 40 = 1 point, antecedent pregnancies: term = 2 points, interval from index pregnancy > 12 months = 4 points, pretreatment b-HCG > 10^5^ m IU/mL = 4 points, largest tumor size including uterus > 5 cm = 2 points, site of metastases: lungs = 0 points, number of metastases < 4 = 1 point, previous failed chemotherapy: none = 0 points)

**Fig. 14:**
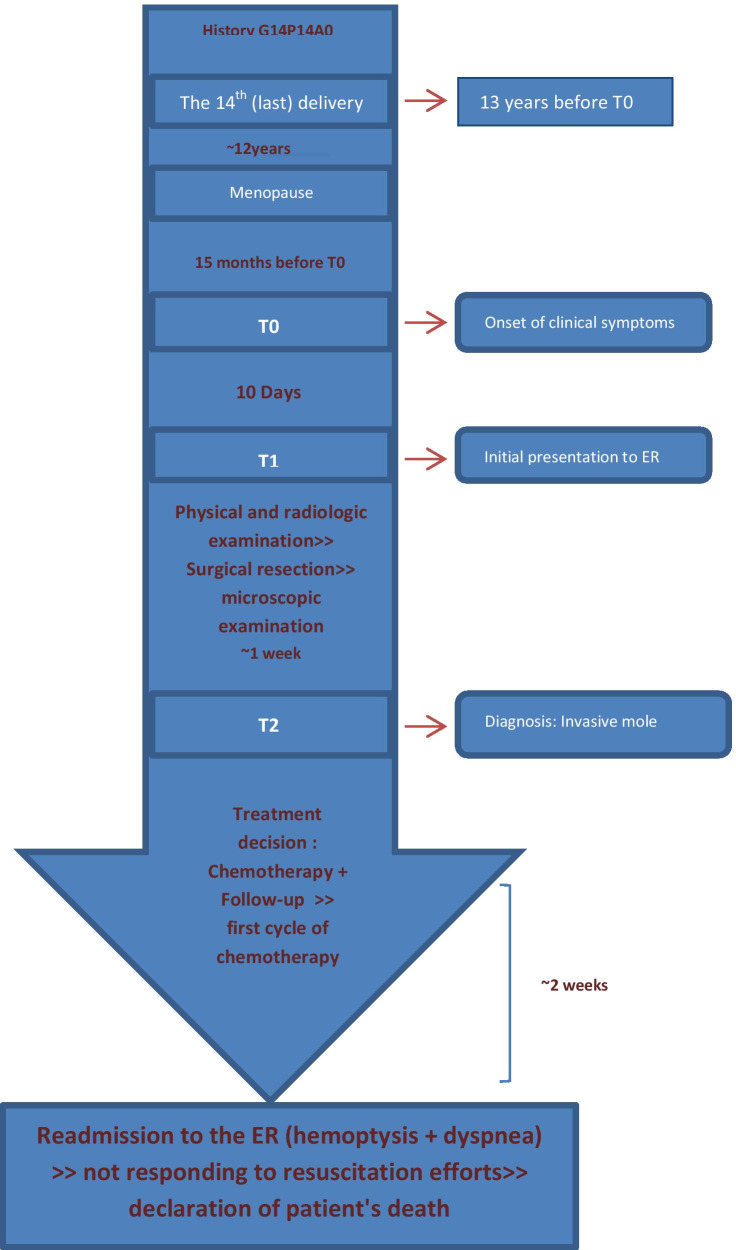
A timeline of the patient’s case

Following surgical resection, the patient was scheduled for combination chemotherapy including methotrexate, etoposide and actinomycin with monitoring of b-HCG levels. However, 2 weeks later the patient was readmitted to the emergency department due to severe dyspnea and hemoptysis. Later the patient developed cardiac arrest and unfortunately she died despite resuscitation efforts. A timeline of the patient's case can be seen in Fig. 14. Written informed consent was obtained from the patient's legal guardian.

## Discussion

Invasive mole is a subtype of gestational trophoblastic neoplasms (GTNs) that usually develops from the malignant transformation of trophoblastic tissue after molar evacuation [3]. It occurs in approximately 15–20% of complete hydatidiform moles and less than 1–5% of partial moles. Invasive moles are more common in women of reproductive age [2]. However, our case was reported in a 55-year-old postmenopausal woman.

The pathogenesis of invasive moles is not fully defined. These neoplasms are preceded by hydatidiform moles in approximately 95% of cases. Other risk factors include pregnancies at extreme reproductive age, history of spontaneous abortions, vitamin A deficiency, oral contraceptives, and paternal and environmental factors [4]. As our patient had no history of molar pregnancies or spontaneous abortions, the initial diagnosis of an invasive mole represented a rare entity.

In 1985, Tsukamoto *et al*. reported eight cases of invasive moles from a total of 20 cases of GTNs in women over 50 years of age. However, none of the invasive mole cases were reported in postmenopausal women [3]. Our case represents the first case reported from Syria, and to our knowledge, only seven cases of invasive moles in perimenopausal and postmenopausal women have been reported worldwide since 2003 (Table 1). De la Fouchardière *et al.* reported an unusual invasive mole following a molar pregnancy in a postmenopausal woman after biologically confirmed menopause [5]. The second case was reported by Taskin *et al.* in 2006 [6]. A case was subsequently reported in 2015, three cases were reported in 2016 [7–10], and the seventh case was reported in 2019 by Martinez Leocardio *et al.* [4].

**Table 1:** Reported cases in the literature of invasive moles in perimenopausal and postmenopausal women since 2003

Author	Year	Age (years)	b-HCG (mU/mL)	Size (cm)	Metastases	Treatment
de la Fouchardière *et al.* [5]	2003	51	26,000	8 cm	Lung + ovaries	Surgery + chemotherapy
Taskin *et al.* [6]	2006	53	84,000	15 cm	None	Surgery
von Welser *et al.* [7]	2015	51	374,875	9.5 cm	None	Surgery
Akyol A *et al.* [8]	2016	51	> 200,000	28 cm	Lung	Surgery + chemotherapy
Guèye M *et al.* [9]	2016	51	Not reported	13 cm	None	Surgery
Nakashima A *et al.* [10]	2016	50	225,000	Not reported	Lung	Surgery + chemotherapy
Martinez Locardio C *et al.* [4]	2019	53	684,180	14 cm	Lung, vagina	Surgery + chemotherapy

*b-HCG* beta-human chorionic gonadotropin

According to their reports, uncontrolled vaginal bleeding and enlargement of the uterus are the most common clinical symptoms in invasive moles, as in our case. Clinical diagnosis requires the presence of these symptoms with persistent elevation of b-HCG levels after molar evacuation [11]. However, in our case, it was a significant challenge to consider GTNs as a differential diagnosis in a postmenopausal woman with no history of molar pregnancies.

Transabdominal ultrasonography (USG) is considered the first-line imaging method for the initial diagnosis of GTNs and for demonstrating the characteristic vesicular appearance. It is also reliable for monitoring processes in patients with elevated serum b-HCG [12]. However, transvaginal USG is considered to have higher specificity in differentiating pelvic masses, while computed tomography (CT) and magnetic resonance imaging (MRI) are recommended for the staging process and detecting metastatic lesions, as in our case [13]. Other radiological techniques include Doppler USG, which can be useful in demonstrating vascular flow within a mass, while the role of fluorine-18-2-fluoro-2-deoxy-d-glucose positron emission tomography/computed tomography (FDG-PET/CT) is mostly limited to identification of individuals at risk of developing GTNs following previous molar pregnancies [11, 13].

Invasive mole is considered a malignant tumor due to its aggressive behavior of local destruction, invasion of the myometrium and the adjacent structures, and the high rate of metastasis (30% at the time of initial presentation). The most common site of metastasis is the lung (30%), followed by the vagina (30%), the liver (10%), and less commonly the brain, bones and the breast [11, 14]. Thus, imaging of the lungs is recommended for all patients. Also, patients with pulmonary metastases have a high risk of developing central nervous system and abdominal metastases, which highlights the role of abdominal CT and cerebral MRI in the staging process [13].

In most cases, invasive moles are diagnosed and treated based on clinical findings. However, histological examination is essential in challenging cases with no typical history, as in our case. The pathological diagnosis of invasive moles is challenging due to the difficulties in differential diagnosis and comparing morphological features [14]. Makovitzky *et al.* reported a case in 2009 of an invasive mole labeled with immunohistochemical staining. The trophoblasts revealed strong positivity for inhibin/activin subunits, Ki67, p53 and glycodelin A [15]. However, their results were built on a single case, and further studies are needed to evaluate the immunohistochemical profiling of invasive moles.

Invasive moles have a high risk of transforming into choriocarcinoma, which is considered the first differential diagnosis of this neoplasm. Choriocarcinomas can be differentiated by multiple morphological features including the absence of villi, high cytologic atypia, and atypical pattern of syncytiotrophoblasts and cytotrophoblasts. Other differential diagnoses include malignant mixed Müllerian tumors (carcinosarcomas), which can easily be excluded by the absence of the biphasic growth pattern of malignant mesenchymal and epithelial components [16, 17]. In our case, the existence of hydropic villi surrounded by a circumferential proliferation of trophoblastic cells invading the myometrium along with radiological features was crucial in confirming the diagnosis of an invasive mole despite the lack of ancillary immunohistochemical and molecular techniques at our institution.

Notwithstanding the aggressive behavior and high metastatic rate of invasive moles, they are highly sensitive to chemotherapy, which is considered the first-line treatment, while surgical procedures might be recommended in specific cases including postmenopausal patients and invasive moles with uncontrolled vaginal bleeding [7, 10, 14].

The chemotherapy regimen is typically designed according to the International Federation of Gynecology and Obstetrics (FIGO) anatomic staging system [18]. Low-risk patients with FIGO scores less than 7 are administered a single agent (methotrexate) as first-line chemotherapy. Actinomycin D can be added in cases of poor response to methotrexate. In cases of high-risk patients (FIGO score > 7), a multi-agent regimen is prescribed (EMA-CO: etoposide, methotrexate, actinomycin D, cyclophosphamide, vincristine) followed a week later by cyclophosphamide and vincristine [Oncovin]), with regular monitoring of serum b-HCG levels [14, 18]. In our case, the patient was a postmenopausal woman with massive vaginal bleeding, and was classified as a high-risk patient (stage III: an invasive mole with pulmonary metastases, risk factor score: 14). Thus a multi-agent chemotherapy regimen (etoposide, methotrexate, actinomycin) was prescribed following hysterectomy and bilateral salpingo-oophorectomy. However, although invasive moles are highly sensitive to chemotherapy, there have been a few case reports highlighting the possibility of sudden death due to rapid disease progression. Ohki *et al*. reported a case of a severe life-threatening hemothorax induced by methotrexate in a metastatic invasive mole [19]. Cauhan *et al.* reported another case of a metastatic invasive mole with sudden death [20]. Unfortunately, life-threatening hemoptysis and dyspnea led to the death of our patient despite resuscitation efforts after the first cycle of chemotherapy.

## Conclusion

Although invasive moles have rarely been reported in postmenopausal women, we managed to confirm the diagnosis of a metastatic invasive mole despite the absence of a relevant history of trophoblastic diseases. We also aimed to highlight the importance of clinical correlation, detailed diagnosis and the appropriate procedure, with subsequent careful follow-up due to the possibility of rapid progression and subsequent mortality despite significant response to chemotherapy.

## Data Availability

Not applicable.

## Abbreviations

GTNs: Gestational trophoblastic neoplasms
GTDs: Gestational trophoblastic diseases
H&E: Hematoxylin and eosin
CT: Computed tomography
USG: Ultrasonography
MRI: Magnetic resonance imaging
b-HCG: Beta-human chorionic gonadotropin
